# Root-Derived Short-Chain Suberin Diacids from Rice and Rape Seed in a Paddy Soil under Rice Cultivar Treatments

**DOI:** 10.1371/journal.pone.0127474

**Published:** 2015-05-11

**Authors:** Haishi Ji, Yuanjun Ding, Xiaoyu Liu, Lianqing Li, Dengxiao Zhang, Zichuan Li, Jingling Sun, Muhammad Siddique Lashari, Stephen Joseph, Yuanduo Meng, Yakov Kuzyakov, Genxing Pan

**Affiliations:** 1 Institute of Resource, Ecosystem and Environment of Agriculture, Nanjing Agricultural University, Nanjing, Jiangsu, 210095, China; 2 Discipline of Chemistry, University of Newcastle, Callaghan, NSW 2308, Australia; 3 National Agricultural Technical Extension and Service Center, Beijing, 100026, China; 4 Department of Soil Science of Temperate Ecosystems, University of Göttingen, Büsgenweg 2, 37077, Göttingen, Germany; 5 Department of Agricultural Soil Science, University of Göttingen, Büsgenweg 2, 37077, Göttingen, Germany; 6 Institute of Environmental Sciences, Kazan Federal University, 420049, Kazan, Russia; 7 Research Center of Terrestrial Ecosystem Carbon Sink and Land Remediation, Zhejiang Agro-Forestry University, Linan, Hangzhou, 311300, China; Institute for Sustainable Plant Protection, C.N.R., ITALY

## Abstract

Suberin-derived substituted fatty acids have been shown to be potential biomarkers for plant-derived carbon (C) in soils across ecosystems. Analyzing root derived suberin compounds bound in soil could help to understand the root input into a soil organic carbon pool. In this study, bound lipids were extracted and identified in root and topsoil samples. Short-chain suberin diacids were quantified under rice (*Oryza sativa* L.) and rape (*Brassica campestris*) rotations with different cultivar combinations in a Chinese rice paddy. After removal of free lipids with sequential extraction, the residual bound lipids were obtained with saponification and derivatization before analysis using gas chromatography–mass spectrometry (GC-MS). Diacids C16 and C18 in bound lipids were detected both in rice and rape root samples, while diacids C20 and C22 were detected only in rape root samples. Accordingly, diacids were quantified in both rhizosphere and bulk soil (0–15 cm). The amount of total root-derived diacids in bulk soil varied in a range of 5.6–9.6 mg/kg across growth stages and crop seasons. After one year-round rice-rape rotation, root-derived suberin diacids were maintained at a level of 7–9 mg/kg in bulk soil; this was higher under a super rice cultivar LY than under a hybrid cultivar IIY. While concentrations of the analyzed diacids were generally higher in rhizosphere than in bulk soil, the total diacid (DA) concentration was higher at the time of rape harvest than at rice harvest, suggesting that rape roots made a major contribution to the preservation of diacids in the paddy. Moreover, the net change in the concentration and the ratios of C_16:0_ DA to C_18:1_ DA, and of C_16:0_ DA to C_18:0_ DA, over a whole growing season, were greater under LY than under IIY, though there was no difference between cultivars within a single growth stage. Overall, total concentration of root-derived suberin diacids was found to be positively correlated to soil organic carbon concentration both for bulk soil and rhizosphere. However, the turnover and preservation of the root suberin biomolecules with soil property and field conditions deserve further field studies.

## Introduction

Lipids from plant sources are a significant source of carbon (C) input to soil [1,2]. Characterizing free or bound fractions of lipids can help elucidate soil organic matter (SOM) dynamics with land use and management changes [3–5]. Lipid compounds are a group of apoplastic biopolymers with tissue–specific composition in the cell walls of endodermis and exodermis and of periderms such as wound periderm [6]. However, suberin is an analogous bio–polyester, containing polyphenolic and polyaliphatic compounds, that is found mostly in root tissues [7]. Furthermore, suberin–derived substituted fatty acids (SFA) could be preserved after incorporation into soil organic matter in soil aggregates surrounding roots [8], owing to their relatively long carbon chains and molecular hydrophobicity. Thus, suberin compounds have been considered a potential tracer to track C input from plant roots and root-C turnover in soil across various ecosystems [8–10].

The aliphatic domain of suberin has been shown to be composed mostly of carboxylic acids, ω–hydroxy carboxylic acids, α, ω–dicarboxylic acids (diacids, DAs) and homologous mid–chain di–hydroxy or epoxy derivatives [11]. Among these monomers, ω-hydroxy fatty acids are not used as root biomarkers, though they are generally considered to be the most prominent substance class [12], for example, for wheat and maize roots [13]. With the development of extraction and quantification procedures [14], diacids of suberin fatty acids, as root derived biomolecules in bound lipids, have been characterized for exploring SOM dynamics across a wide range of soils [10,13]. Above-ground tissues of grain crops, often removed after harvest, are less suberized than that of woody plants [13] and thus contribute less to SOM in agricultural soils. Instead, diacids extracted from soil may better represent crop root-derived biomolecules for assessing root input to SOM. Furthermore, the aliphatic molecules of suberin diacids could exert higher stability in soil than lignin compounds [8,15]. Therefore, analysis of these diacids in soil could provide important information about crop root-derived C to the stable pool of SOM in agricultural soils.

Rice (*Oryza sativa* L.) is cultivated on 155 million hectares of world croplands, occupying approximately 14% of all global arable land [16]. Although rice production on paddy soils has been considered an important source of biogenic greenhouse gases [17], paddies, with improved management [18,19], could have higher SOM storage and greater C sequestration potential than they currently have. As one of the major cultivated soil types in China [20], rice paddy soils have high soil organic carbon storage via carbon sequestration from root input carbon [21] through enhanced physical protection [22] and chemical binding with oxyhydrates of Fe/Al [23]. Recently, molecular recalcitrance [24] and biological stabilization [25] have also been suggested for SOM accumulation and stabilization in rice paddies. Over the last few decades, many studies have shown higher increases in SOM in paddy soils than in dry croplands of China [19,26] and fast accumulation of SOM in soils recently converted to rice cultivation [27]. The newly accumulated SOM observed in bulk soil has often been credited for an increase in physically protected SOM in micro-aggregates [27]. However, there have been few studies reporting the abundance of root-derived biomolecules as related to root-C input from rice and rotated crops, and their relative contributions to SOM in rice paddies [28].

Super hybrid rice (super rice in short) is a varietal type of rice that combines different plant varieties with heterosis and achieves super high yields through hybridization between *indica* and *japonica* [29,30]. Super rice cultivars are characterized by higher root biomass and higher leaf-area index [31] with enhanced photosynthesis and biomass production. This could, in turn, potentially increase root-C input via rhizodeposition of photosynthesized carbon hydrates. While changes in SOM after continuous cultivation of super rice have been discussed [32], there is no field study addressing root-C contribution to rice soil via root activity in field conditions.

This study’s objective is to demonstrate the abundance and composition of suberin diacids as root-derived biomolecules of rice and the rape crops, and to characterize the changes in crop-growth stages in a rice paddy under continuous super rice breeding rotated with rape. The root suberin diacids were analyzed using a modified extraction and GC-MS detection assay. Concentrations of the suberin diacids were compared both between bulk and rhizosphere soil samples and between rice cultivars in order to infer potential difference in root-derived C input into paddy soil under rice-rape rotation.

## Materials and Methods

### Ethics Statement

In this study, no specific permission was required either for the soil/plant sampling at the site or for the biomolecular analysis at the laboratory. No endangered or protected species were involved in the field work.

### Experimental site and crop cultivation

Field work of soil and plant sampling was conducted in a demonstration farm for high yield rice breeding, located in Jianhua village (117°47'E, 31°39'N), Zhonghan Town, Anhui Province, China. The farm was under management by the Rice Research Institute, Anhui Academy of Agricultural Sciences, China. The paddy soil was a Hydroagric Stagnic Anthrosol [33] derived from lacustrine deposits. The experiment with super rice breeding was established in 2005 with continuous cultivation of super rice cultivars in comparison with hybrid rice. The rice paddy had been cultivated under summer rice (*Oryza sativa* L.) and winter rape seed (*Brassica campestris*) rotation in recent decades. For summer rice, a super rice cultivar belonging to the LY series and a conventional hybrid rice belonging to the ⅡY series had been cultivated, while for winter rape, two parallel cultivars, both belonging to the QY series, were cultivated in a year round rotation. The experimental design of this study is shown in Table 1. Each cultivar treatment was conducted in triplicate and the plots were arranged in a randomized complete block design.

**Table 1:** Cultivars design of rice and rape cultivation of 2011–2012 used in this study.

	Cultivar Treatments	
Crop cultivation	A	B
Summer rice	LY (Super rice)	ⅡY (Hybrid rice)
Winter rape	QY 10	QY 19

For the samples used in this study, rice was sown on May 10, 2011 and transplanted with a spacing of 30 cm between rows and 13.3 cm in rows on June 11, 2011. Hybrid and super rice cultivars were fertilized with 180 and 210 kg N/ha of urea, 33 and 39 kg P/ha of superphosphate, and 62 and 124 kg K/ha of potassium chloride, respectively. While phosphorus fertilizer of superphosphate was applied as base fertilizer only, total N was split by 50%, 20% and 30%, respectively, before sowing, tillering and spiking, for both cultivars. Meanwhile, 60% of potassium chloride was applied as basal fertilizer and the other 40% was applied at the spiking stage. The soil moisture regime was managed with a scheme of flooding before spiking and waterlogged intermittently thereafter until harvest. Plant protection was consistent across the cultivars. Rape seed was transplanted after rice harvest with a spacing of 33.3 cm between rows and 25.0 cm in rows, totaling 120,000 plants/ha. Rape seed cultivars were fertilized with the same amount of 210 kg N/ha, with a ratio of 1:0.43:0.71 (N:P:K) across the two cultivars.

### Collection and treatment of soil and root samples

In this study, soil samples were collected before, during and after crop growth for analysis of root suberin diacids as well as for soil physico-chemical analysis. Specifically, a sampling of initial bulk topsoil (0–15cm) was done after rape harvest, on the 25^th^ of May 2011, before a new rotation year of crop production in 2011–2012. Over the course of the rice growing season, both bulk soil and rhizosphere samples were collected in each cultivar plot at the end of tillering (8^th^ July of 2011), at grain filling stage (5^th^ September of 2011), and at harvest (13^th^ October of 2011). Meanwhile, both bulk soil and rhizosphere samples under rape cropping were also collected at harvest (26^th^ May of 2012). Rhizosphere samples were obtained from the crop root zone by shaking and washing the soil attached to crop roots, following a procedure described in detail by Butler *et al*. [34] and by Wiesenberg *et al*. [35]. The remaining root was preserved as root samples. Bulk samples of topsoil, however, were collected randomly in a spacing area between the plants. For all these samples, sampling was done in random triplicate in order to form a composite sample for a single treatment plot.

After being shipped to the laboratory, any plant detritus and gravel was removed from the collected samples. After air–drying in room temperature, the soil samples were ground and passed through a 2 mm sieve. The root samples were washed and then oven–dried at 60°C. A portion of soil and root samples was further ground with a stainless steel grinder to pass through a 0.15 mm sieve for root suberin diacids assay and SOM-C analysis.

### Analysis of soil properties

Soil basic properties were measured following the standard protocols described by Lu [36]. To summarize, soil pH was measured with a glass electrode using a 1:2.5 soil/water ratio with a precision pH meter (Mettler Toledo Seveneasy, Switzerland). SOM concentration was measured using wet digestion and oxidation with potassium dichromate. Total nitrogen was analyzed using the micro Kjeldahl method. Total phosphorus was digested with perchloric acid and sulfuric acid, and analyzed with colorimetry (TU–1810) at 700 nm. Particulate organic matter (POM) was measured according to the procedure described by Cambardella and Christensen [37] as follows: Dispersing 20 g dried soil (<2 mm) in a solution of 100 mL 0.5 mol/L NaOH, and filtering the suspension through a 53 μm sieve. The material remaining on the sieve was analyzed for carbon with a CNS analyzer (Variomax CNS Analyzer, German Elementar Company, 2003). Dissolved organic matter (DOM) was extracted with CO_2-_free distilled water at 25°C, and then measured using a TOC analyzer (Jena Multi N/C 2100) [32]. Microbial biomass carbon (MBC) was determined using the fumigation–extraction method [38]. The cation exchange capacity (CEC) was measured with the ammonium acetate (1 mol/L, pH 7.0) leaching method. Concentration of soil particles was determined using a hydrometer after dispersion with 0.5 mol/L NaOH as described by Zhou *et al*. [39]. Basic properties and organic matter pools of topsoil are presented in Tables 2 and 3, respectively.

**Table 2:** Basic soil properties (0–15 cm) under rice cultivars after rice harvest in 2011.

Cultivar	pH (H_2_O)	CEC (cmol/kg)	Total N (g/kg)	Total P (g/kg)	Sand (%)	Clay (%)
LY	6.51±0.18a	22.71±0.98a	2.21±0.14a	0.69±0.12a	20.43±0.53b	46.10±1.26a
ⅡY	6.45±0.19a	20.37±0.48b	2.02±0.10a	0.57±0.08a	26.04±2.14a	45.03±0.84a

LY, super rice cultivar; ⅡY, hybrid rice cultivar. CEC, cation exchange capacity.

Different lowercase characters indicate significant difference at *p*<0.05.

**Table 3:** Soil organic matter pools of bulk soil (0–15cm) under the two cultivars after rice harvest in 2011.

Cultivars	SOM-C (g/kg)	POM-C (g/kg)	DOM-C (mg/kg)	MBC (mg/kg)
LY	23.29±1.54a	5.40±0.20a	164.70±5.04a	390.72±14.71a
ⅡY	21.30±1.21a	4.70±0.65b	149.25±5.48b	311.43±16.43b

LY, super rice cultivar; ⅡY, hybrid rice cultivar. SOM-C, POM-C and DOM-C, measured in carbon of total, particulate and dissolved organic matter; MBC, microbial biomass carbon.

Different lowercase characters indicate significant difference between cultivars at *p*<0.05.

### Root suberin diacids analysis

#### 1. Processing of soil samples

Soil samples were prepared for root suberin diacids analysis as follows:

Step 1, Delipidation: Before depolymerisation of suberin, a portion of a soil sample (<0.149 mm) was extracted three times using a 1:10 w/v dichloromethane–methanol (2:1, v/v) solution in room temperature for 10 hours in order to remove free lipids [40,41]. The extract was separated from soil particles by centrifugation (3500 rpm, 15 min), and the supernatant was decanted.

Step 2, Saponification: Soil residues obtained in Step 1 were depolymerized to release suberin compounds. The residues obtained were shaken for 22 hours in an aqueous solution of potassium hydroxide (1.0 M KOH) in methanol under a N_2_ atmosphere [13,42].

Step 3, Derivatization: The product of saponification was trans-esterified by a 12% BF_3_–MeOH solution under a N_2_ atmosphere at 75°C for 0.5 h, and the residual BF_3_ solution was quenched with water [43]. The solution was filtered before the residue was washed using a methanol solution (methanol/water 9:1, v/v) [13].

Step 4, Extraction: The filtrate from Step 3 was combined with 4 mL saturated NaCl solution, and subsequently extracted with dichloromethane three times to obtain hydrophobic monomers [40,44]. The extraction was then washed with 0.5 mol/L NaCl solution three times, and the pH was adjusted to 7.0 with KOH solution. After the supernatant was decanted, the residue was combined with anhydrous Na_2_SO_4_ and dried in room temperature [45] prior to GC analysis.

#### 2. Processing of root samples

The ground root samples were soaked for 10 hours and then washed thoroughly with a mixture of chloroform and methanol (2:1, v/v). The residues were extracted with chloroform in a Soxhlet extractor for 48 hours. After air–drying in room temperature, they were treated for 16 hours with a mixture of cellulase (5 g/L) and pectinase (1 g/L) in a 0.05 M acetate buffer (pH 4) at 30°C with rotatory shaking at 220 rpm in a Thermostatic Oscillator (HZ–9211KB, Shanghai, China). Subsequently, they were washed thoroughly with distilled water and then dried in an oven at 60°C. The above–mentioned procedure was repeated twice. The final residues were washed with a mixture of chloroform and methanol (2:1, v/v) and then dried in room temperature [46].

Finally, the saponification, derivatization, extraction and drying of the root samples for suberin diacids analysis were conducted using the same procedure as for the soil samples.

### GC–MS and GC analysis

#### 1. Qualitative analysis

The dried extracts were dissolved in a solution of pyridine or n–heptane and filtered through a 0.45 μm filtration membrane, before being analyzed by gas chromatography–mass spectrometry (GC–MS).

The GC–MS analysis involved an HP–5 capillary column (HP–5 MS 5% Phenyl Methyl Silox: 939.53782, 325°C: 30 m × 250 μm × 0.25 μm film thickness) with Helium carrier gas at 34 mL/min constant flow and in an oven that was kept at 60°C for 2 min, then programmed to shift from 60°C to 310°C at 10°C/min, and then held for 2 minutes at 310°C. Samples were injected in split mode (30:1 ratio at 280°C injector temperature). Being more soluble than n–heptane in preparation experiments, pyridine was used as a supplementary solvent for extraction of the suberin fatty acids [45]. Finally, a mixture of pyridine and n–heptane was used as solvent for quantitative analysis with GC to improve the extraction in this study.

#### 2. Quantitative analysis

Trans–esterified monomers dissolved in the solution of n–heptane mixed with pyridine were separated with an Agilent 7890 gas chromatograph (GC) which was equipped with an Agilent 19091J–413 HP–5 5% Phenyl Methyl Siloxan column (J&W Scientific, CA, USA; 30 m × 320 μm × 0.25 μm). The GC oven was kept at 60°C for 1 min, then programmed to increase from 60°C to 220°C at 40°C /min, subsequently from 220°C to 280°C at 3°C /min, and finally from 280°C to 325°C at 15°C/min. Trans-esterified monomers were quantified with a flame ionization detector (FID) coupled to the GC, using Nonadecanoic methyl ester (NU–CHEK PREP, INC) dissolved in pyridine as an internal standard [13,47]. Four external calibrations were used of the hexadecane 1, 16–dioic acid (C_16:0_ DA) (TCI–EP), octadecane 1, 18–dioic acid (C_18:0_ DA) (Dr. Ehrenstorfer), eicosanedioic 1, 20–dioic acid (C_20:0_ DA) (Alfa Aesar) and docosane 1, 22–dioic acid (C_22:0_ DA) (Sigma–Aldrich), which were all trans-esterified by the same procedure used for each soil sample. The protocol used did not allow a reliable detection of the other fatty acid monomers such as ω -hydroxy fatty acids.

The sensitivity of GC analysis was very high as the detection limit could be as low as 10^–12^ g. The concentration of suberin diacids C_16:0_ DA, C_18:1_ DA, C_18:0_ DA, C_20:0_ DA and C_22:0_ DA in the tested samples was all up to a level of at least 10^–10^ g. The recovery of C_16:0_ DA, C_18:0_ DA, C_20:0_ DA and C_22:0_ DA was 76.55 ± 27.41%, 100.89 ± 21.77%, 94.17 ± 10.06% and 77.97 ± 23.44%, while the coefficient of variation (CV) of C_16:0_ DA, C_18:1_ DA, C_18:0_ DA, C_20:0_ DA and C_22:0_ DA, was 5.10%, 13.84%, 2.80%, 17.77% and 23.77%, respectively.

### Data processing and statistics

Data were expressed as means plus/minus standard deviation (mean ± SD). The difference between cultivars was examined using one–way analysis of variance (ANOVA) while the difference between rhizosphere and bulk soil samples was examined using a paired *t*–test. Bivariate correlation analysis was performed to find the relationship between diacids and SOM-C. A significant difference or correlation was defined at *p*<0.05. All statistical analyses were carried out using SPSS 16.0.

## Results

### Basic soil properties and organic matter pools under cultivars

As shown in Table 2, there were similar physico-chemical properties of the paddy soil after continuous rice cultivation across the cultivar treatments. However, there were significantly higher concentrations of POM (by 15%), DOM (by 10%) and MBC (by 25%) under cultivars LY than under II Y. In contrast, no significant difference in total SOM was seen between cultivars LY and II Y (Table 3).

### Monomers of root suberin diacids detected in root and rhizosphere samples

**Fig 1:**
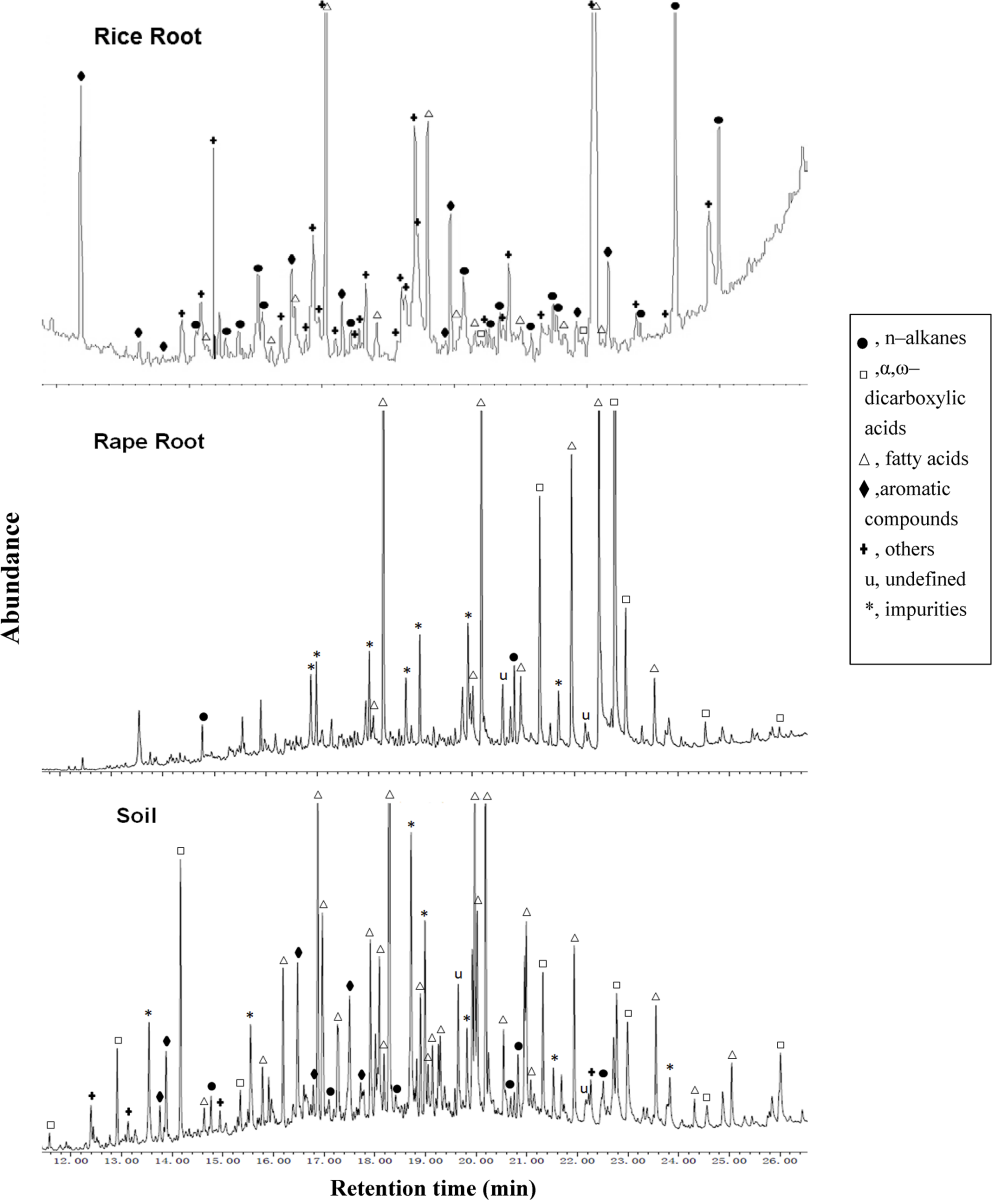
GC–MS chromatograms of the major molecules extracted from rice root, rape root and soil.

Four major classes of organic compounds; alkanes, fatty acids, diacids and aromatic compounds, were identified in the bound lipids both of rhizosphere and root samples (Fig 1 and S1 Table). Here, the significant difference in aromatic compound abundance between rape and rice samples could be due to the difference in the solvent used. Dissolution was conducted for rape in n–heptane only and for rice root in the mixture of n–heptane and pyridine. Fatty acids were dominant in soil samples, alkanes dominated in rice roots, while fatty acids and diacids were predominant in rape roots. With this method, ω-hydroxy fatty acids were not able to be detected. Dissolution of root suberin compounds in the mixture allowed successful detection of the diacids for rice though dissolution in n–heptane, without pyridine, was also successful for rape roots.

Furthermore, the major root biomolecules under the cultivated rice were the short-chain diacids of C16 and C18, similar to those found in study [12]. Being indicative of rice root-input biomolecules, monomers of hexadecanedioic acid (C_16:0_ DA), octadecenedioic acid (C_18:1_ DA) and octadecanedioic acid (C_18:0_ DA) were all detected in the rice roots. Besides these diacids, low levels of longer-chain monomers of eicosanedioic acid (C_20:0_ DA) and docosanedioic acid (C_22:0_ DA), typically from rape plants, were also detected in rape root and soil samples but not in rice roots. Thus, the modified procedure was able to detect the root-derived biomolecules both for rice and rape, though not the ω-hydroxy fatty acids monomers, in the rice paddy under rice and rape rotation (S1 and S2 Figs).

### Root suberin diacids under rice cropping

**Fig 2:**
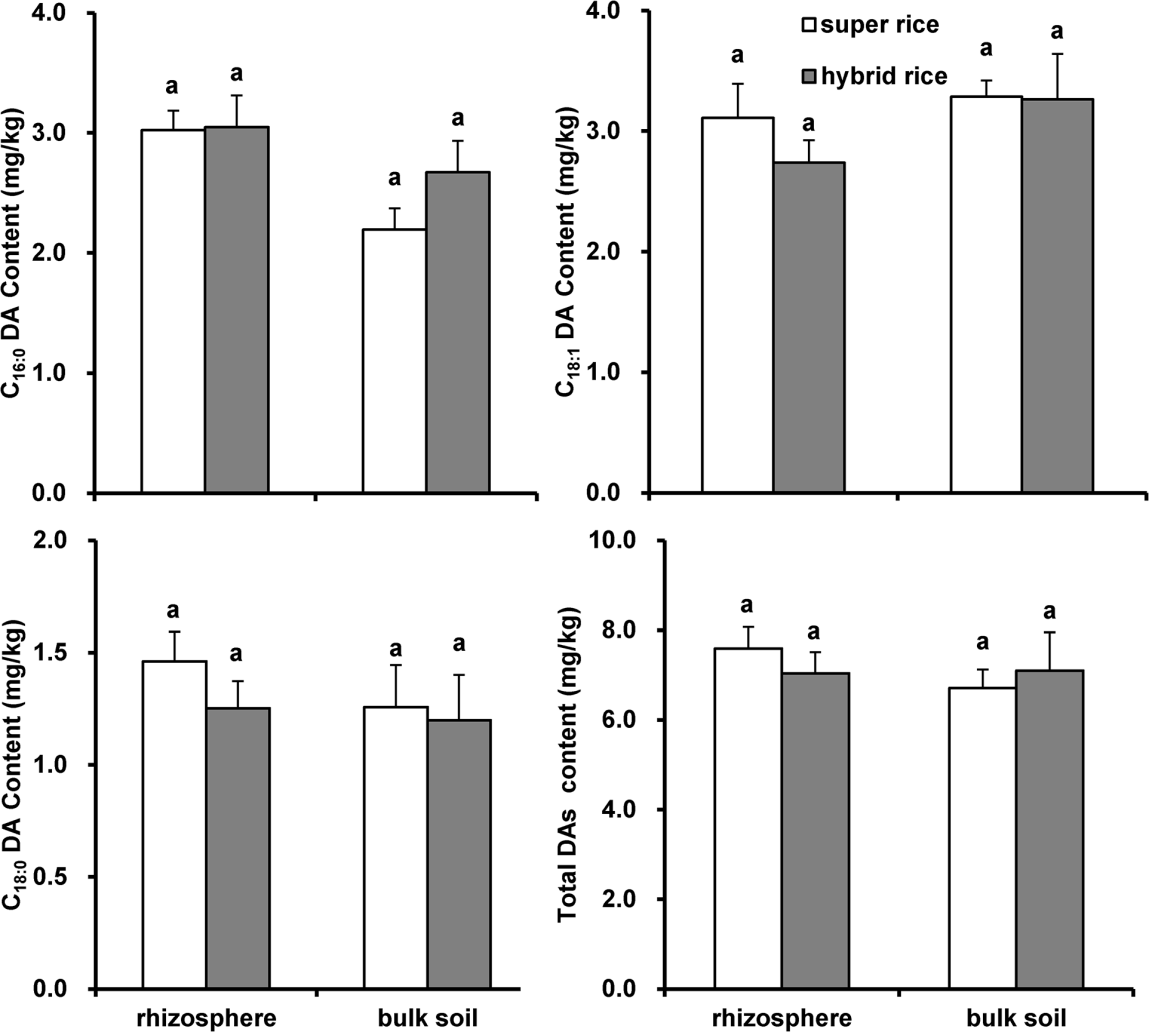
Root suberin diacid concentrations (mean ± SE, n = 9) of rhizosphere and bulk soils under rice calculated on average of the measurements across the growing stages. Total DAs: concentration of all monomers of ΣC_16:0_ DAs, C_18:1_ DA and, C_18:0_ DA. Different low case letters represent significant difference at *p*<0.05 between bulk and rhizosphere samples.

Concentrations of root suberin diacid monomers (ΣC16–18), calculated by averaging the measurements across the growing stages, ranged from 1.0 mg/kg to 8.0 mg/kg of dry soil and were generally higher in rhizosphere than in bulk soil samples (Fig 2). Notably, concentrations of C_16:0_ DA and total DAs were significantly higher in rhizosphere than in bulk soil under super rice (LY) while no significant difference occurred under hybrid cultivars (II Y). More root suberin diacids were preserved in rhizosphere samples under super rice LY than under the hybrid rice.

Concentrations of the diacids and SOM-C, across rice growing stages in bulk soil and rhizosphere samples, are presented respectively in Table 4 and Table 5. While no consistent changes in the concentrations of different monomers were observed across the growing stages, there was a slight decrease in the total concentration of diacids (ΣC16-C18) in the bulk soils, as the crops approached the ripening stage. Compared to concentrations before transplantation, the diacids monomers generally decreased at the filling stage and increased again at harvest, both for bulk soil and rhizosphere samples. There were no significant differences between rice cultivars in total DAs concentration and in different monomers both for bulk soil and rhizosphere samples except in the monomer of C_16:0_ DA in bulk soil. Here, a net change over a whole rice-growing season was calculated and assessed. This showed a net change in concentration of C_16:0_ DA and of total DAs, that was only slight and insignificant (-1.0 mg/kg and -1.3 mg/kg) under LY but significant (-2.0 mg/kg and -3.0 mg/kg) under IIY. However, for the rhizosphere samples, there was an insignificant change of 1.0 mg/kg and 1.3 mg/kg respectively of root suberin C_16:0_ DA and total DAs under IIY.

**Table 4:** Concentration of SOM-C (g/kg) and root suberin diacids (mg/kg) and their ratios in bulk soil under rice across growing stages.

Growth stage	Cultivar	SOM-C	C_16:0_ DA	C_18:1_ DA	C_18:0_ DA	Total DAs	C_16:0_ DA/C_18:1_ DA	C_18:1_ DA/C_18:0_ DA	C_16:0_ DA/C_18:0_ DA
Before transplantation	LY	25.54±1.76A	3.33±0.53A	3.69±0.88A	1.34±0.41A	8.36±1.36A	0.93±0.15A	3.03±1.20B	2.77±1.10A
	ⅡY	27.74±0.27a	4.12±0.47a	4.01±1.39a	1.44±0.84a	9.57±1.60a	1.09±0.29a	3.52±1.94a	3.87±2.75a
Tillering	LY	24.76±2.09AB	2.70±0.40B	3.20±0.53A	1.64±0.52A	7.53±1.33A	0.85±0.12AB	2.06±0.51B	1.73±0.32B
	ⅡY	26.52±0.00a	3.29±0.53ab	4.27±0.88a	1.63±0.61a	9.18±2.02a	0.78±0.04a	2.71±0.48a	2.11±0.47a
Filling	LY	25.04±1.32AB	1.61±0.21C	3.35±0.70A	0.62±0.11B	5.58±0.51B	0.51±0.17C	5.63±2.18A	2.65±0.50A
	ⅡY	22.73±0.29b	2.86±0.81b	2.96±1.10a	0.71±0.04a	6.54±1.86a	0.99±0.09a	4.21±1.76a	4.06±1.34a
Harvest	LY	23.29±1.54B	2.28±0.41B	3.31±0.84A	1.51±0.44A	7.10±1.60A	0.71±0.13B	2.23±0.28B	1.58±0.35B
	ⅡY	21.30±1.21b	2.13±0.35b	2.80±0.76a	1.63±0.65a	6.56±1.73a	0.78±0.09a	1.77±0.35a	1.38±0.29a
Change after rice cropping	LY	-2.25±2.56*	-1.06±0.57*	-0.38±0.94	0.18±0.56	-1.26±1.92	-0.22±0.14	-0.81±0.65*	-1.19±0.68*
	ⅡY	-6.44±1.21	-1.98±0.35	-1.21±0.76	0.19±0.65	-3.01±1.73	-0.31±0.09	-1.74±0.35	-2.49±0.29

LY, super rice cultivar;ⅡY, hybrid rice cultivar. Different capital and lowercase letters in a single column represent significant difference at *p*<0.05 between stages respectively for LY and IIY cultivars. Significant difference between cultivars only in content of C_16:0_ DA, at all stages except at harvest.

**Table 5:** Concentration of SOM-C (g/kg) and root suberin diacids (mg/kg) and their ratios in rhizosphere under rice across growing stages.

Growth stage	Cultivar	SOM-C	C_16:0_ DA	C_18:1_ DA	C_18:0_ DA	Total DAs	C_16:0_DA/C_18:1_DA	C_18:1_DA/C_18:0_DA	C_16:0_DA/C_18:0_DA
Tillering stage	LY	25.50±1.47A	3.03±0.59AB	3.75±0.95A	1.85±0.43A	8.64±1.47A	0.84±0.21B	2.03±0.23A	1.69±0.43B
	ⅡY	26.94±1.30a	2.55±0.40a	2.86±0.47a	1.06±0.25a	6.46±1.12a	0.90±0.01a	2.73±0.18a	2.44±0.18a
Filling stage	LY	25.18±2.32A	2.56±0.42B	2.20±0.79B	1.10±0.19B	5.87±0.94B	1.27±0.43A	2.08±1.00A	2.38±0.54A
	ⅡY	22.75±1.67a	3.04±0.92a	2.51±0.54a	1.41±0.40a	6.95±1.79a	1.21±0.20a	1.81±0.14a	2.18±0.36a
Harvest stage	LY	25.52±1.62A	3.47±0.44A	3.37±0.63A	1.43±0.40B	8.28±1.05A	1.06±0.23AB	2.42±0.46A	2.56±0.67A
	ⅡY	24.07±2.47a	3.57±0.04a	2.96±0.58a	1.22±0.28a	7.75±0.26a	1.23±0.25a	2.56±1.07a	3.01±0.66a
Change after rice cropping	LY	0.03±1.46*	0.44±0.65	-0.38±1.06	-0.42±0.42	-0.36±1.14	0.22±0.39	0.39±0.41	0.87±0.84
	ⅡY	-2.87±2.47	1.02±0.04	0.11±0.58	0.16±0.28	1.29±0.26	0.34±0.25	-0.17±1.08	0.58±0.67

LY, super rice cultivar:ⅡY, hybrid rice cultivar. Different capital and lowercase letters in a single column represent significant difference at *p*<0.05 between stages respectively for LY and IIY cultivars. No significant difference in concentration of the root suberin diacids between cultivars in a single stage.

### Root suberin diacids under rape cropping

**Fig 3:**
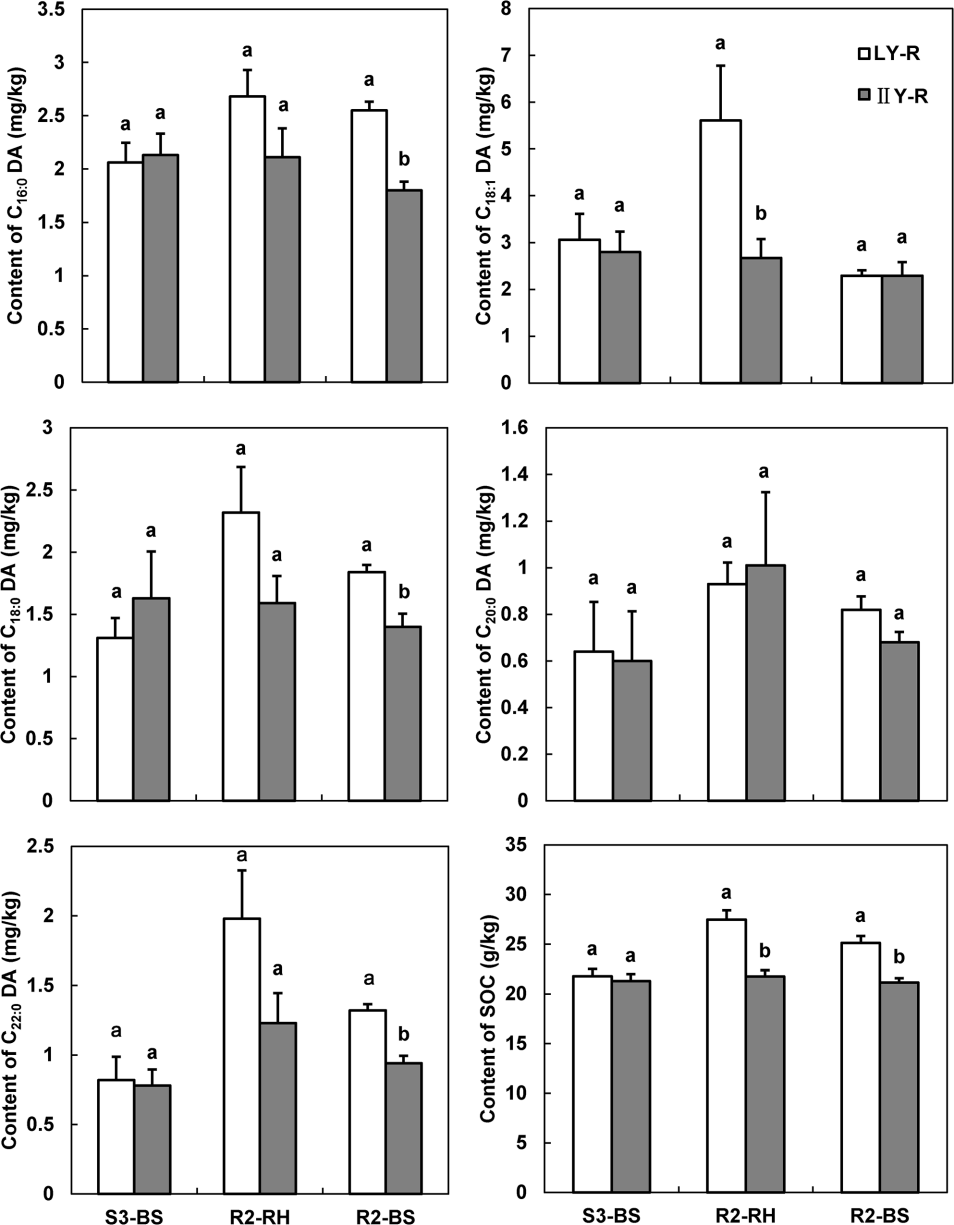
Root biomarker concentrations and soil organic matter measured in carbon (mean ± SE, n = 3) in the soil under rape seed. S3, before transplantation of rape seed (after harvest of the rice), R2, at harvest of rape seed; RH, the rhizosphere soil; BS, bulk soil; LY, super rice; ⅡY, hybrid rice. LY–R, super rice-rape rotation; ⅡY–R, hybrid rice-rape rotation. Different letters represent different statistical class among the cultivars at *p*<0.05.

Under rape cropping the soil contained a wide range of monomers of root suberin diacids viz. C_16:0_ DA, C_18:1_ DA, C_18:0_ DA, C_20:0_ DA and C_22:0_ DA. Data of the concentrations of these monomers and SOM in bulk soil before rape transplantation (S3), and both in the rhizosphere and bulk soil at rape harvest, (R2), are illustrated in Fig 3. The concentrations of all monomers detected varied in a range of 1.2–5.7 mg/kg for rhizosphere and of 0.8–2.5 mg/kg for bulk soils. After rape cropping, the diacid concentrations generally increased greatly in the rhizosphere and moderately in bulk soils, compared to the bulk soil after rice cropping. Similar to the overall trend under rice (Fig 2), the root suberin diacid concentrations under rape were generally higher in the rhizosphere than in bulk soil. There were generally significant differences in the concentrations of most monomers detected between cultivar treatments. In particular, the concentration of C_22:0_ DA, a potential rape root biomarker, was much greater (by up to 40%) both in bulk soil and rhizosphere, in LY plots than in IIY plots. Therefore, higher concentrations of root suberin diacids were preserved after rape cropping under super rice (LY) than under hybrid cultivar (IIY).

## Discussion

### Variation of root suberin diacids with rice growing stages and cropping seasons

Using the modified protocol with GC-MS, the concentration of root suberin diacids could be successfully determined both in the bulk soil and in the rhizosphere. The data presented above show the differences in the concentration of different monomers, and total diacids between rhizosphere and bulk samples at the different rice growing stages as well as for rape cropping. The protocol used did not allow the measurement of ω-hydroxy acids, which were generally considered to be shoot-derived plant aliphatic biomolecules [13]. Here, short-chain monomers of C_16:0_ DA and C_18:1_ DA as the major diacids and of C_18:0_ DA as the minor diacid were detected under rice cultivation, in addition to these, longer-chain monomers of C_20:0_ DA and C_22:0_ DA were detected in small quantities under rape cultivation. This is similar to the findings of the study [12] which addresses the different role of the monomers in the transport of water into the plants. The dominance of diacid monomers of C_16:0_ DA and C_18:1_ DA over C_18:1_ DA was also reported for tree-root-derived biomarkers [9]. While there were slight inconsistencies between the cultivars, the diacid concentrations were generally higher in the rhizosphere than in bulk soil, especially after rape cultivation. As relatively fresh OM, root-derived diacids could decompose over time, more rapidly in aerobic conditions under rape cultivation than under intermittent waterlogging with rice cultivation [48]. This could be explained, in part, by a bigger difference in the diacid concentrations between rhizosphere with prominent root input, and bulk soil without prominent root input, under rape cropping. In addition, much higher diacid concentrations in the soil at the rape harvest compared to at the rice harvest, suggests a major contribution by rape roots to the soil storage of root-derived suberin diacids under rice-rape rotation.

Data in Table 5 exhibit hardly any difference in rhizosphere concentrations of the root suberin diacids across rice growing stages. Furthermore, no significant differences were observed in bulk soil samples except in the concentration of monomer C_16:0_ DA across all stages. This lack of difference was in contrast to the findings by Mendez-Millan *et al*. [13] that root-derived suberin aliphatic compounds were different both in isotopic and monomers composition between C3 (wheat) and C4 (maize) plants. Wiesenberg *et al*. [5] found that there was a difference in the composition of carboxylic acids, especially the ratio of n-C24 versus n-C22+26 and their respective C-isotope values, for different types of plant roots. Similarly, when calculating the ratios of different monomers, we found differences in ratios of monomer C_16:0_ DA to C_18:1_ DA and of C_16:0_ DA to C_18:0_ DA between different stages, especially between tillering and harvest stages for cultivar LY both in the bulk and rhizosphere samples. This corresponded to the difference in concentration of monomer C_16:0_ DA between the stages. This finding suggested that the composition pattern of the diacids could have changed with growing stages. There were certainly differences in field soil properties, nutrient status and other soil conditions between the rice growing stages with different rice production managements. In a study [35] using both C3 and C4 plants, the distribution pattern of plant lipid biomolecules was not affected by short term artificial pulse ^14^CO_2_ labelling but rather it was sensitive to environmental changes. Therefore, there could be considerable differences in the composition patterns with the rice, though differences between cultivars were insignificant.

### Relationship between root suberin diacids and soil organic carbon

**Fig 4:**
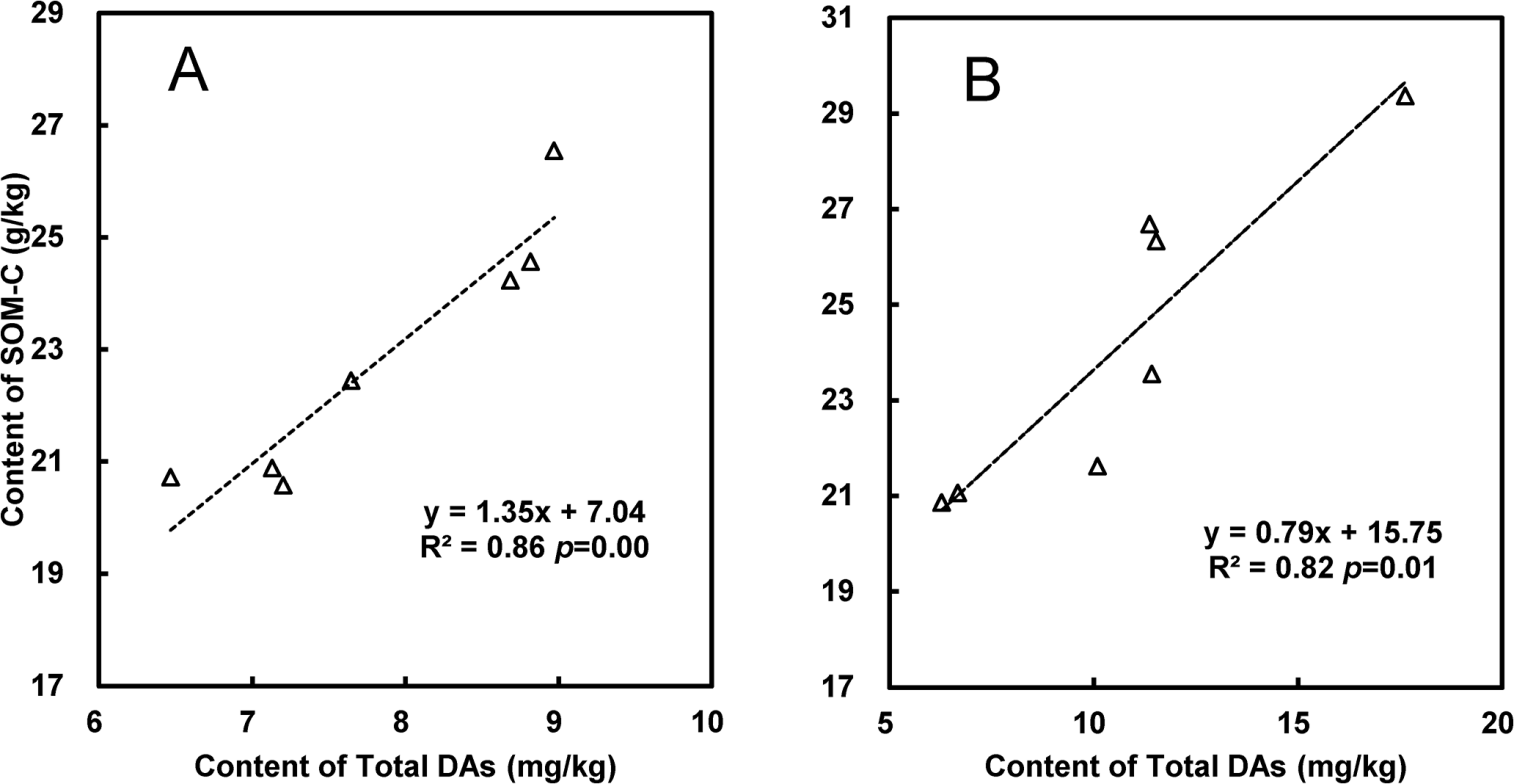
The relationship between concentrations of total diacids and of SOM-C of bulk soil (0–15 cm) (A) and of rhizosphere (B) after rape seed harvest. Total DAs, total concentrations summarized of ΣC_16:0_ DA, C_18:1_ DA, C_18:0_ DA C_20:0_ DA and C_22:0_ DA.

In this study, concentrations of root suberin diacids of C_16:0_ DA, C_18:1_ DA, C_18:0_ DA, C_20:0_ DA and C_22:0_ DA, and SOM concentrations both of bulk soil and rhizosphere across the crop growing stages followed a pseudo–normal distribution as shown by 2–tailed single series Kolmogorov–Smirnov Test. There was a significant positive correlation between total DAs and SOM-C concentration both for bulk soil and rhizosphere throughout a whole rice–rape rotation year (Fig 4). This correlation could indicate SOM-C changes in the rice paddy over the cultivar treatment plots in relation to their root-derived organic carbon input. Organic matter accumulation in soils has often been shown to be associated with the preservation of hydrophobic organic matter, mostly in the form of lignin and lipids [49] including the suberin fatty acids analyzed here. Hydrophobic organic matter is known to indicate SOM sequestration from crop straw input in paddy soils rich in free oxyhydrates of Fe/Al [50]. Nierop *et al*. [51] argued that ester–linked macromolecules such as cutin and suberin could be used as stable indicators of plant–derived organic matter in soils. Moreover, a greater portion of bound lipids to total SOM shows SOM stability, as bound lipids have been shown to be more stable than bulk SOM [15].

The significant correlation of diacids with SOM-C observed here could suggest that root suberin biomolecules of diacids are an important component of plant-derived OM contributing to C storage in soil. Here, we compared the changes in the concentrations of the different monomers after rice cropping and found that the concentration of C_16:0_ DA and the ratio of C_16:0_ DA to C_18:0_ DA decreased less, corresponding to a smaller decrease in SOM-C, under cultivar LY than under IIY (Tables 4 and 5). Meanwhile, a significant increase in POM-C of bulk soil was observed after rice harvest under LY compared to under IIY (Table 3). An increase in POM could represent an increased input of plant-derived organic matter under super rice cultivation, because POM has been generally considered to be relatively fresh organic matter [32]. Meng *et al*. [32] reported that continuous cultivation of super rice resulted in a reduction in indigenous SOM. However, in their study, a higher percentage of the coarse sand micro-aggregates (2–0.2 mm in size) were found under continuous super rice cultivation compared to under hybrid rice [32]. Presumably, more root-derived C in the soil under the super rice-rape rotation had been preserved compared with the hybrid rice-rape rotation. The increase in POM under super rice LY here coincided with these findings, indicating higher preservation of fresh organic input from rice roots under LY than under IIY.

Furthermore, DOM-C and MBC concentrations in bulk soil at rice harvest were also higher under super rice cultivar LY than under hybrid rice IIY, suggesting relatively high available C substrate under the super rice cultivar (Table 3). It has been well known that microbial growth could be greatly promoted with fresh organic matter input [52], which often induces increased soil respiration and C turnover in agricultural soils [53]. While total SOM-C was not observed as being significantly reduced under LY relative to IIY, the increase in fresh organic matter under super rice did not appear to compromise the potential for enhanced C mineralization in the short term (5 years of continuous super rice breeding in this site) [32]. It is still not clear if the change in SOM concentration with super rice cultivation could lag behind the change in the labile SOM pools such as POM, DOM as well as MBC. In this study, however, the concentrations of C_16:0_ DA and C_22:0_ DA diacids as root derived biomolecules were very significantly and positively correlated with SOM-C in bulk soil (*p* = 0.009, 0.022) and in the rhizosphere (*p* = 0.043, 0.013). Nevertheless, as there was no net increase in the total concentrations of the root suberin diacids over a whole rice growing season, the mechanism of the preservation of these root-derived suberin diacids in the rice soil and their long term fate needs further study.

## Conclusions

Root suberin diacids were quantified both in bulk and rhizosphere samples in paddy soil under different cultivars across a whole rice and rape rotation year. While a difference in the monomer concentrations was hardly found between cultivar treatments in a single stage, the composition pattern of the monomers was altered with rice growing stages under cultivar LY. Higher concentrations of rice root suberin diacids were generally observed in rhizosphere than in bulk soil, and after rice cropping, there was a smaller decrease in them under a super rice cultivar LY than under a hybrid cultivar IIY. This change was in line with the changes in labile organic matter pools in the bulk soil. Significant correlations of these root suberin diacids with soil organic matter both in bulk soil and rhizosphere could suggest the potential for fresh root-derived organic matter accumulation in the rice paddy. However, the turnover and preservation of the root suberin biomolecules with soil property and field conditions are still not clear from short-term cultivation, and this deserves further field studies.

## Supporting Information

S1 Table Compounds identified in base hydrolysis products of rice and rape root, and soil. (DOCX)

S1 Fig GC-MS diagram of root biomarkers for both rice and rape derived diacids species. (TIF)

S2 Fig GC-MS diagram of rape specific root biomarkers. (TIF)
