# Supplementary material for: Root-Derived Short-Chain Suberin Diacids from Rice and Rape Seed in a Paddy Soil under Rice Cultivar Treatments
Source: PLoS One. 2015 May 11;10(5):e0127474. doi: 10.1371/journal.pone.0127474 (PMC4427476; doi:10.1371/journal.pone.0127474)
Supplement: S1 Table — (DOCX) [file pone.0127474.s001.docx]

Table S1. Compounds identified in base hydrolysis products of rice root, rape root and soil

| Rice root (n-heptane and pyridine) |  |
| --- | --- |
| R.T. Area % ID Prob. % | R.T. Area % ID Prob. % |
| **Alkane 18.01** | 22.475 0.22 13-Tetradecen-1-ol acetate 94 |
| 13.570 0.35 Pentadecane 96 | 23.551 0.30 Docosanoic acid, methyl ester 95 |
| 14.432 0.21 Heptadecane, 2-methyl- 76 | 25.045 0.43 Tetracosanoic acid, methyl ester 98 |
| 14.768 1.05 Hexadecane 97 | **α,ω-Dicarboxylic acids 5.40** |
| 15.493 0.60 Tetradecane, 2,6,10-trimethyl- 78 | 6.764 0.19 2-Butenedioic acid (E)-, dimethylester 95 |
| 15.975 1.35 Dodecane, 2,6,11-trimethyl- 96 | 8.755 0.53 Butanedioic acid, methoxy-, dimethyl ester 91 |
| 16.384 1.08 Hexadecane,  2,6,10,14-tetramethyl- 86 | 12.918 0.17 Octanedioic acid, dimethyl ester 81 |
| 17.100 1.30 1-Octadecene 74 | 14.169 0.76 Nonanedioic acid, dimethyl ester 91 |
| 17.426 1.00 Heptadecane 91 | 21.321 1.46 Hexadecanedioic acid, dimethyl ester 93 |
| 17.640 0.72 Octadecane, 2-methyl- 93 | 22.776 2.29 Octadecenedioic acid dimethyl ester |
| 17.728 1.13 Heptadecane, 3-methyl- 83 | 22.991 Octadecanedioic acid, dimethyl ester 83 |
| 18.477 0.61 1-Octadecene 90 | **Aromatic compounds 18.88** |
| 18.638 0.82 Nonadecane 90 | 13.886 0.22 Benzoic acid, |
| 19.261 0.88 1-Nonadecene 93 | 4-hydroxy-3-methoxy-, methyl ester 95 |
| 19.544 0.75 Octadecane 92 | 16.491 8.70 2-Propenoic acid, |
| 19.592 0.63 Octadecane 95 | 3-(4-hydroxyphenyl)-, methyl ester 98 |
| 19.680 0.81 2-Methyl-Z-4-tetradecene 91 | 16.783 0.92 Benzoic acid, |
| 20.586 0.74 Eicosane 96 | 4-hydroxy-3,5-dimethoxy-, hydrazide 86 |
| 20.829 1.88 Docosane 94 | 17.513 6.26 2-Propenoic acid, |
| 21.686 1.05 Tricosane 95 | 3-(4-hydroxy-3-methoxyphenyl)-, methyl ester 99 |
| 22.514 0.73 Tetracosane 98 | 18.521 0.62 .alpha.-d-Glucofuranosyl benzenesulfonate 95 |
| 23.307 0.14 Pentacosane 98 | **Others 5.79** |
| 25.790 0.24 2,6,10,14,18,22-Tetracosahexaene, 98 | 12.397 0.42 Vanillin 97 |
| **fatty acids 11.85** | 13.200 1.55 Citric acid, trimethyl ester 90 |
| 16.189 1.31 Methyl tetradecanoate 89 | 15.293 0.68 1-Chloroeicosane 81 |
| 18.283 5.81 Hexadecanoic acid, methyl ester 98 | 16.686 0.78 Sulfurous acid, butyl hexadecyl ester 89 |
| 19.972 1.54 9-Octadecenoic acid, (Z)-, methyl ester, 98 | 18.419 1.28 Tetradecane 1-bromo- 93 |
| 20.191 1.97 Octadecanoic acid, methyl ester 99 | 19.373 1.08 E-15-Heptadecenal 93 |
| 21.934 0.27 Eicosanoic acid, methyl ester 96 |  |
|  | **Others undefined 21.70** |

| Rape root (n-heptane) |  |
| --- | --- |
| R.T. Area% ID Prob. % | R.T. Area% ID Prob. % |
| **Alkane 1.93** | **α,ω-Dicarboxylic acids 28.89** |
| 14.768 0.55 Hexadecane 97 | 21.321 4.95 Hexadecanedioic acid, dimethyl ester 95 |
| 20.829 1.38 Docosane 95 |  |
| **fatty acids 43.02** | 22.781 19.98 Octadecenedioic acid dimethyl ester |
| 18.093 0.45 cis-9-Hexadecenoic acid 93 |  |
| 18.283 10.69 Hexadecanoic acid, methyl ester 97 | 22.991 3.37 Octadecanedioic acid, dimethyl ester 93 |
| 19.972 1.13 9-Octadecenoic acid (Z)-, methyl ester 99 |  |
| 20.021 1.69 9-Octadecenoic acid, methyl ester, (E)- 99 | 24.539 0.59 Eicosanebioic acid, dimethyl ester |
| 20.191 7.42 Octadecanoic acid, methyl ester 99 |  |
| 20.951 1.84 Cyclopentaneundecanoic acid, methyl ester 89 | 25.980 Docosanedioic acid, dimethyl ester 74 |
| 21.939 6.42 Eicosanoic acid, methyl ester 99 | **Others undefined 13.81** |
| 22.475 11.96 9,12-Octadecadienoic acid (Z,Z)- 93 |  |
| 23.551 1.42 Docosanoic acid, methyl ester 99 |  |
|  |  |
| Soil (n-heptane and pyridine) |  |
| R.T. Area % ID Prob. % | R.T. Area % ID Prob. % |
| **Alkanes 5.00** | **α,ω-Dicarboxylic acids 9.11** |
| 14.768 0.27 Hexadecane 97 | 11.579 0.10 Heptanedioic acid, dimethyl |
| 17.100 0.51 Octadecane 91 | ester 91 |
| 18.414 0.61 Pentadecane, 3-methyl- 70 | 12.918 0.73 Octanedioic acid, dimethyl |
| 18.638 0.33 1-Nonadecene 78 | ester 93 |
| 19.213 0.34 1-Nonadecene 80 | 14.169 2.07 Nonanedioic acid, dimethyl |
| 20.581 0.56 Eicosane 68 | ester 95 |
| 20.829 0.92 Docosane 95 | 15.347 0.39 Decanedioic acid, dimethyl |
| 22.514 0.72 Tetracosane 98 | ester 91 |
| 23.307 0.37 Pentacosane 83 | 21.321 1.65 Hexadecanedioic acid, dimethyl ester 95 |
| 24.558 0.37 Cyclotetracosane 80 | 22.777 2.18 Octadecenedioic acid dimethyl ester |
| **fatty acids 44.37** | 22.991 1.99 Octadecanedioic acid, dimethyl ester 55 |
| 14.636 0.24 Methyl 11-methyl-dodecanoate 96 | 24.539 Eicosanebioic acid, dimethyl ester 94 |
| 15.785 0.63 Tridecanoic acid, 12-methyl-, methyl ester 98 | 25.980 Docosanedioic acid, dimethyl ester 76 |
| 16.189 1.47 Methyl tetradecanoate 98 | **Aromatic compounds 6.25** |
| 16.876 3.27 Methyl 13-methyltetradecanoate | 13.760 0.33 Phenol, 2,4-bis(1,1-dimethylethyl) 96 |
| (C15 iso-FAME) 97 | 13.882 0.81 Benzoic acid, |
| 16.968 2.27 Methyl 13-methyltetradecanoate | 4-hydroxy-3-methoxy- , methyl ester  96 |
| (C15 anteiso-FAME) 86 |  |
| 17.270 1.44 Pentadecanoic acid, methyl ester 95 | 13.843 0.10 Butylated Hydroxytoluene 95 |
| 17.913 1.92 Pentadecanoic acid, | 16.477 1.70 2-Propenoic acid, |
| 14-methyl-, methyl ester 98 | 3-(4-hydroxyphenyl)-, methyl ester 98 |
| 18.093 1.80 9-Hexadecenoic acid, methyl |  |
| ester, (Z)- 99 | 16.788 0.54 Benzoic acid, |
| 18.185 0.98 11-Hexadecenoic acid, methyl ester 97 | 4-hydroxy-3,5-dimethoxy-,hydrazide 98 |
| 18.288 8.35 Hexadecanoic acid, methyl ester 99 | 17.504 2.28 2-Propenoic acid, 3-(4-hydroxy-3-methoxyphenyl)-, |
| 18.906 1.30 Heptadecanoic acid, methyl ester 97 | methyl ester 99 |
| 19.052 0.79 cis-10-Heptadecenoic acid, methyl ester 96 | 17.786 0.49 Phthalic acid, isobutyl |
| 19.140 1.06 11-Hexadecenoic acid, | octadecyl ester 72 |
| 15-methyl-, methyl ester 91 | **Others 5.00** |
| 19.257 0.75 Hexadecanoic acid, | 12.397 0.39 Vanillin 97 |
| 14-methyl-, methyl ester 98 | 13.132 0.26 Methyl 3-hydroxy-decanoate |
| 19.295 0.99 Heptadecanoic acid, | hydroxy acid 90 |
| 14-methyl-, methyl ester 91 | 14.948 0.18 Z-6-Tetradecen-1-ol acetate 80 |
| 19.972 3.07 9-Octadecenoic acid (Z)-, methyl ester 99 | 17.757 0.29 2-Piperidinone, |
| 20.021 2.46 9-Octadecenoic acid, methyl ester, (E)- 99 | N-[4-bromo-n-butyl ]- 70 |
| 20.191 3.92 Octadecanoic acid, methyl ester 99 | 17.723 0.54 Hexadecane, 1-(ethenyloxy)- 91 |
| 20.547 0.94 Octadecanoic acid, 10-methyl-, | 18.059 0.50 Heptadecanenitrile 81 |
| methyl ester, (R)- 94 | 22.226 0.50 |
| 20.990 2.05 7-Hexadecenoic acid, methyl ester, (Z)- 95 | 4,8,12,16-Tetramethylheptadecan-4-olide 93 |
| 21.082 0.62 Nonadecanoic acid, methyl ester 94 | 22.265 0.74 9-Octadecenamide, (Z)- 83 |
| 21.939 1.87 Methyl 18-methyl nonadecanoate 99 | 22.480 0.48 13-Tetradecen-1-ol acetate 92 |
| 23.551 1.27 Docosanoic acid, methyl ester 99 | 26.009 1.12 Methyl 2-hydroxy-tetracosanoate 93 |
| 24.310 0.31 Tricosanoic acid, methyl ester 99 |  |
| 25.045 0.60 Tetracosanoic acid, methyl ester 98 |  |
|  | **Others undefined 7.56** |
