# Supplementary figures and images for: Root-Derived Short-Chain Suberin Diacids from Rice and Rape Seed in a Paddy Soil under Rice Cultivar Treatments
Source: PLoS One. 2015 May 11;10(5):e0127474. doi: 10.1371/journal.pone.0127474 (PMC4427476; doi:10.1371/journal.pone.0127474)

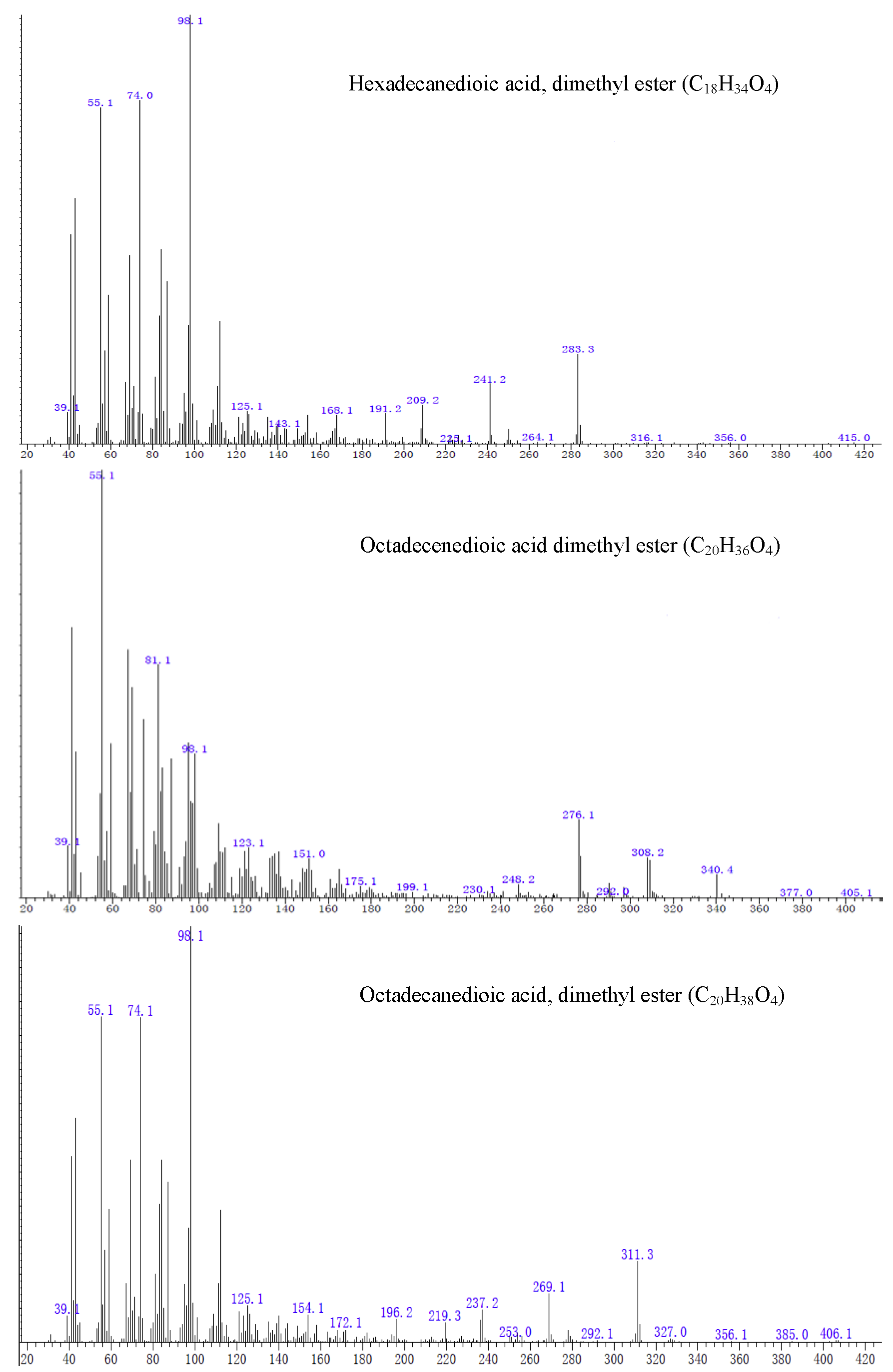

Supplement: S1 Fig — (TIF) [file pone.0127474.s002.tif]

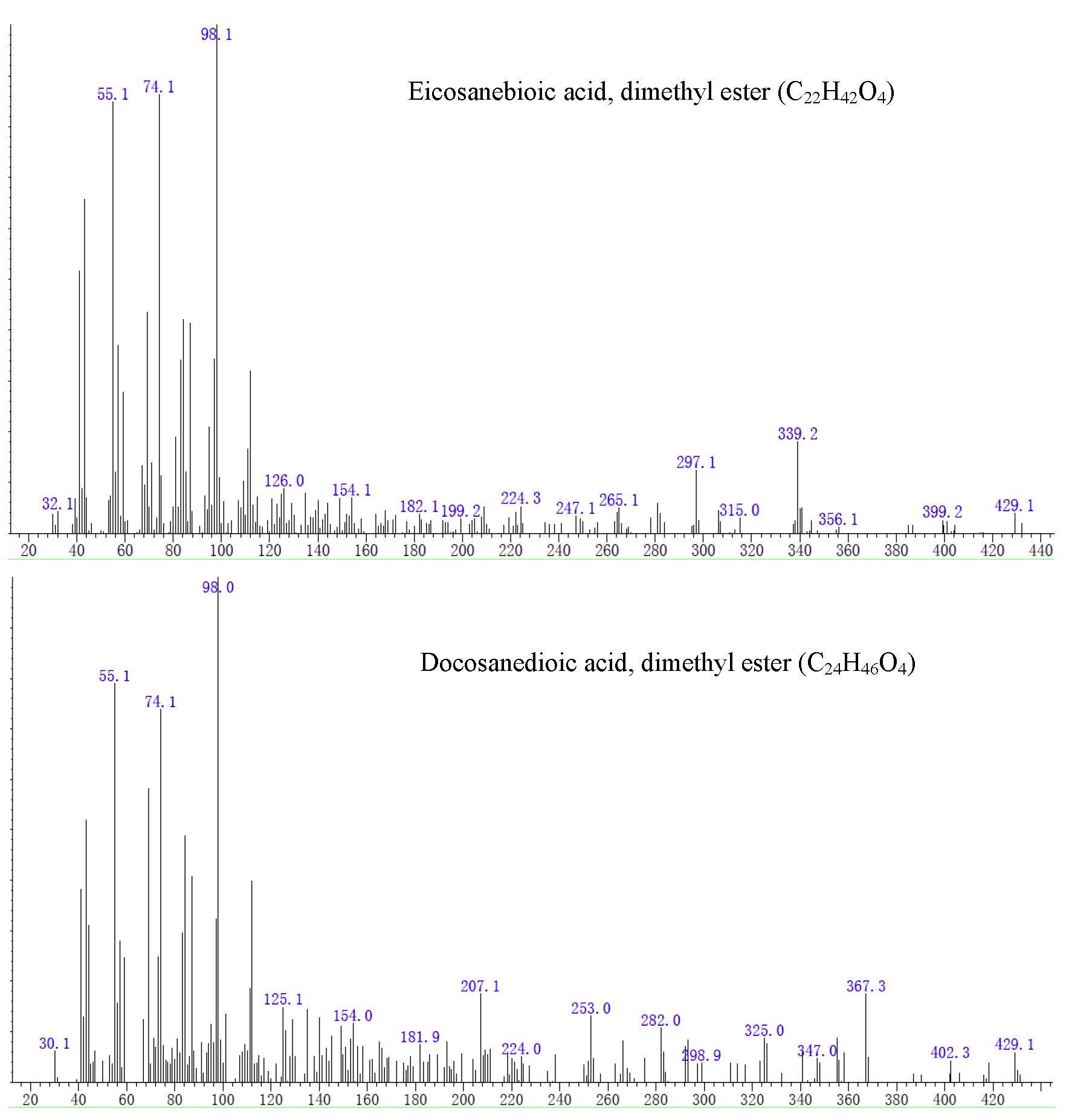

Supplement: S2 Fig — (TIF) [file pone.0127474.s003.tif]
